# Local Effects of Astringents upon the Blood Vessels

**Published:** 1878-07

**Authors:** A. Rosenstein


### ARTICLE XI.

### *Investigations on the Local Effects of the so-called Astringents upon the Blood Vessels.*

Dr. A. Rosenstein, (*Wuerzburg, Phys. Med. Verhdlg.*, 1876,) examined the effects of solutions of argentum nitricum, plumbi acetas, acidum tannicum, gallicum and pyrogallicum, ferrum sesquichloratum and alumen, by applying them to the mesentery of curarized frogs, and measuring the calibre of the affected vessels with the micrometer. The most powerful contraction was produced by nitrate of silver in a solution of one to ten per cent., the observations being often disturbed by the ensuing partial opacity of the tissues. The contraction in many cases involved one-half of the lumen, both of the arteries and veins, being less marked in the capillaries, and manifesting itself in the course of a few seconds. R. observed a stoppage of the circulation in the affected vessels, which, was permanent in the capillaries, but at times only transitory in the arteries and veins. Tannic acid, contrary to expectations, was found to have the opposite effect, dilating arteries, veins and capillaries, as much as one-half of their calibre, while they became at the same time choked with blood corpuscles. The dilated vessels immediately contracted on the application of nitrate of silver. Gallic and pyrogallic acids were found to have the same effect as tannic acid. Acetate of lead produced a contraction of the arteries and veins, though less markedly than nitrate of silver. Its effect could not be traced to the capillaries. Occasionally, a stoppage of the circulation was observed. The vessels almost invariably contained white coagula, consisting of conglomerated, colorless blood corpuscles, often adhering to the walls of the vessels, and thus giving to their transverse sections a beaded appearance. A ten per cent. solution of liquor ferri sesquichlorati had no perceptible effect. A fifty per cent. solution caused a contraction of the vessels, though in a still lower degree than acetate of lead. This contraction was limited to the arteries and veins, while the capillaries remained dilated. A frequent result was coagulation and discoloration of the blood within the vessels. A discrepancy was observed in the results of the various experiments with alum solution. The vessels were in some cases contracted, in others dilated; while in others again, no appreciable change was noticed. In the capillaries, especially the smaller ones, the circulation often ceased. In order to prevent reflex action, he extirpated the spinal column of the frog, and destroyed the communication between the vessels and the heart, without changing in any way the local effect of the substances above mentioned. From the results of these experiments the author infers that only nitrate of silver and acetate of lead can be said to exert an astringent action, *i. e.*, to cause contraction of the tissues, this effect being of uncertain occurrence in alum, and the liq. ferri sesquichlor., and entirely absent in the tannic group.—*Toledo Med. and Surg. Journal*

---

